# Impact of Sickle Cell Trait and Naturally Acquired Immunity on Uncomplicated Malaria after Controlled Human Malaria Infection in Adults in Gabon

**DOI:** 10.4269/ajtmh.17-0343

**Published:** 2017-12-18

**Authors:** Bertrand Lell, Benjamin Mordmüller, Jean-Claude Dejon Agobe, Josiane Honkpehedji, Jeannot Zinsou, Juliana Boex Mengue, Marguerite Massinga Loembe, Ayola Akim Adegnika, Jana Held, Albert Lalremruata, The Trong Nguyen, Meral Esen, Natasha KC, Adam J. Ruben, Sumana Chakravarty, B. Kim Lee Sim, Peter F. Billingsley, Eric R. James, Thomas L. Richie, Stephen L. Hoffman, Peter G. Kremsner

**Affiliations:** 1 Centre de Recherches Médicales de Lambaréné (CERMEL), Lambaréné, Gabon;; 2 Institut für Tropenmedizin, Eberhard Karls Universität Tübingen and German Center for Infection Research (DZIF), Tübingen, Germany;; 3 Sanaria Inc., Rockville, Maryland;; 4 Protein Potential, LLC, Rockville, Maryland

## Abstract

Controlled human malaria infection (CHMI) by direct venous inoculation (DVI) with 3,200 cryopreserved *Plasmodium falciparum* sporozoites (PfSPZ) consistently leads to parasitemia and malaria symptoms in malaria-naive adults. We used CHMI by DVI to investigate infection rates, parasite kinetics, and malaria symptoms in lifelong malaria–exposed (semi-immune) Gabonese adults with and without sickle cell trait. Eleven semi-immune Gabonese with normal hemoglobin (IA), nine with sickle cell trait (IS), and five nonimmune European controls with normal hemoglobin (NI) received 3,200 PfSPZ by DVI and were followed 28 days for parasitemia by thick blood smear (TBS) and quantitative polymerase chain reaction (qPCR) and for malaria symptoms. End points were time to parasitemia and parasitemia plus symptoms. PfSPZ Challenge was well tolerated and safe. Five of the five (100%) NI, 7/11 (64%) IA, and 5/9 (56%) IS volunteers developed parasitemia by TBS, and 5/5 (100%) NI, 9/11 (82%) IA, and 7/9 (78%) IS by qPCR, respectively. The time to parasitemia by TBS was longer in IA (geometric mean 16.9 days) and IS (19.1 days) than in NA (12.6 days) volunteers (*P* = 0.016, 0.021, respectively). Five of the five, 6/9, and 1/7 volunteers with parasitemia developed symptoms (*P* = 0.003, NI versus IS). Naturally adaptive immunity (NAI) to malaria significantly prolonged the time to parasitemia. Sickle cell trait seemed to prolong it further. NAI plus sickle cell trait, but not NAI alone, significantly reduced symptom rate. Twenty percent (4/20) semi-immunes demonstrated sterile protective immunity. Standardized CHMI with PfSPZ Challenge is a powerful tool for dissecting the impact of innate and naturally acquired adaptive immunity on malaria.

## INTRODUCTION

Controlled human malaria infection (CHMI) is a tool that can be used to accelerate the clinical development of antimalarial interventions.^1^ In addition, it has been used as an immunotherapy for neurosyphilis^2^ and to study the pathobiology of malaria and antimalarial immunity.^3–5^ During the past 90 years, techniques to inoculate parasites were performed—mainly by exposure to the bites of infected mosquitoes, injection/transfusion of parasitized blood, and rarely, injection of parasite-infected mosquito salivary glands. In recent decades, techniques to induce CHMI have become safer, more reproducible, and focused on assessing protective efficacy of vaccine and drug candidates. Most recently, the availability of aseptic, purified, cryopreserved *Plasmodium falciparum* (Pf) sporozoites (SPZ), a product called Sanaria^®^ PfSPZ Challenge has led to further advances in standardization. After direct venous inoculation (DVI) of 3,200 SPZ with PfSPZ Challenge, all malaria-naive participants have been consistently infected.^6,7^ In contrast to previous methods, the dosage, generation, and strain of the parasite are tightly controlled and, due to cryopreservation, clinical studies have fewer requirements for infrastructure and fewer restrictions on timing and design. These advances together with present knowledge and multidimensional techniques to study immunology and metabolism led us, more than five decades following the last experiments,^3^ to assess whether CHMI can be used to study innate resistance and naturally adaptive immunity (NAI) to malaria. For more than 100 years,^8^ it has been known that NAI develops after repeated exposure to Pf.^9^ Nevertheless, our understanding of the underlying mechanisms is incomplete. We have also known that individuals with sickle cell trait have a reduced chance of developing severe malaria. To systematically investigate these mechanisms and identify critical targets that could be used to develop subunit vaccination approaches, a stringent and reproducible infection model with a well-defined and relevant end point is required. We assessed whether standardized CHMI by DVI of 3,200 PfSPZ in healthy, adult, lifelong malaria–exposed volunteers is a suitable model that 1) is safe and well tolerated, 2) results in parasite dynamics different from CHMI in malaria-naive adults, 3) results in parasite dynamics different between HbAA and HbAS carriers, and 4) can be used to assess acquired immunity against the pre-erythrocytic and erythrocytic stages of the Pf lifecycle.

## METHODS

### Study design and participants.

The study was designed to assess the effect of lifelong malaria exposure and sickle cell trait on infectivity and on time to parasitemia and to malaria-related symptoms. Primary end points resulting in treatment were clinical malaria, defined as parasitemia plus symptoms, asymptomatic asexual parasitemia ≥ 1,000 parasites/μL, or Day 28, when all nonpreviously treated study subjects were treated. PfSPZ Challenge was inoculated by DVI in a dosage of 3,200 PfSPZ. The trial was conducted at the *Centre de Recherches Médicales de Lambaréné* (CERMEL) in Lambaréné, Gabon from July 2014 to February 2016. Lambaréné is situated in the Central African rainforest and has a perennial transmission of malaria with reduced transmission in the dry season from July to September.^10^ The indigenous adult population is “semi-immune” against malaria, with no cases of severe malaria and a high prevalence of asymptomatic infections.^11^ The prevalence of sickle cell trait is approximately 15%.^12^ Volunteers were healthy adults between 18 and 30 years of age, without a history of severe chronic diseases and without a history of intake of an antimalarial drug in the preceding weeks. Pregnancy and the intention to become pregnant during the study were exclusion criteria. Volunteers were screened and allocated to one of three groups: nonimmunes (NI group), who were malaria naive or minimally exposed without sickle cell trait (Europeans visiting or living in Gabon); semi-immunes (I) with lifelong malaria exposure, and with hemoglobin HbAA (IA group) or with sickle cell trait (HbAS) (IS group). Malaria naivety was defined as no history of Pf malaria and no long-term (> 5 years) residence in a malaria-endemic area. Lifelong malaria exposure was defined as long-term (> 10 years) residence in a highly malaria-endemic area. A total of 25 volunteers, 10 each for groups IA and IS and five in the NI group were planned to be recruited. These numbers would allow detection of a difference in the time to parasitemia of at least 2 days between the groups (NI<IA<IS) with more than 95% power, assuming low variation in pre-patent period in the semi-immunes. In case that CHMI with 3,200 PfSPZ would not be successful in more than 50% of IA and IS group volunteers, the dose was escalated to 12,800 PfSPZ.

### Procedures.

On screening, volunteers were evaluated for clinical and laboratory abnormalities on two occasions. In addition, hemoglobin status was assessed by gel electrophoresis. After inclusion and allocation, volunteers, including the NI group, started a regimen of 12-hourly 300 mg clindamycin for 5 days (Day 7 to Day 3), a highly efficacious and well-tolerated antimalarial regimen that targets asexual liver and blood stage parasites.^13^ Clindamycin has a half-life of 2–4 hours^14^ and does not interfere with subsequent CHMI. Two days after the last dose of clindamycin, volunteers received 3,200 PfSPZ of PfSPZ Challenge by DVI (Day 0). PfSPZ Challenge consists of aseptic, purified, infectious PfSPZ, strain NF54, produced and cryopreserved according to good manufacturing practices.^15^ PfSPZ Challenge is reconstituted in 0.5 mL buffer and injected through DVI. Volunteers remained on site for at least 1 hour after DVI, were visited on the following day, and contacted daily thereafter. From Day 5 until treatment, volunteers were visited daily to sample blood and record symptoms. Treatment with artemether–lumefantrine was administered to nonimmunes once the thick blood smear (TBS) was positive. In semi-immunes, treatment was administered once malaria was suspected. Malaria was diagnosed after a positive TBS (at any level of parasitemia) and symptoms that could be attributed to malaria, such as headache, fever, and arthralgia were present. Volunteers with parasitemia above 1,000 parasites/μL were treated irrespective of symptoms. All volunteers without parasitemia or remaining at low density parasitemia without symptoms were treated on Day 28. Quantitative thick blood films were prepared as described,^16^ at least once a day. Two or more microscopists were required to observe a minimum of two unambiguous parasites to declare a slide positive. The limit of detection was adjusted to a 95% probability of detecting parasitemia of at least 10 parasites/μL. In addition, 0.5 mL blood sample was collected to measure submicroscopic parasitemia by quantitative polymerase chain reaction (qPCR) using published procedures,^17^ with minor modifications; blood was stored in RNAlater^™^ and extracted using Qiagen blood isolation kits (both Qiagen, Hilden, Germany). The lower limit of quantification of the qPCR was 20 parasites/mL; the lower limit of detection of the qPCR 5 parasites/mL. Gametocytes were detected microscopically and through qPCR of Pfs16 and Pfs25 mRNA.^18^ Genotyping of parasites was performed by nested polymerase chain reaction (PCR) using *msp2* and *glurp* primers and subsequent sequencing of the *msp2* gene.

Antibodies against the Pf circumsporozoite protein (PfCSP), exported protein-1 (PfEXP1), and merozoite protein-1 (PfMSP1) before and on Day 28 after injection of PfSPZ Challenge for CHMI were assessed by enzyme-linked immunosorbent assay as previously described.^19^ The serum dilution at which the optical density (OD) was 1.0 was assessed and designated as ‘OD 1.0’. An individual was considered to have developed antibodies if the difference between the OD 1.0 on Day 28 and Day 0 was at least 50 and the ratio of the OD 1.0 on day 28 to day 0 was at least 3.

Symptoms and adverse events (AEs) were captured through semistructured interviews and diaries, followed by coding using MedDRA. All AEs were graded (mild, moderate, or severe), and their relatedness to the study interventions (clindamycin, PfSPZ Challenge, malaria, or malaria treatment) was assessed on a five-grade scale (definitely not related, unlikely, possibly, probably, and definitely related). Laboratory deviations were graded with a modified toxicity grading scale,^20^ based on reference values from the study area. On Days 35, 84, and 168 after challenge, volunteers were seen for follow-up safety assessments, including safety laboratory tests.

The study received approval by the Gabonese National Ethics Committee (*Comité National d’Ethique de la Recherche*) and was conducted under the US FDA Investigational New Drug application. Before enrollment, written informed consent was obtained from volunteers, and understanding of the study and procedures was assessed with a quiz. The study followed the principles of the Declaration of Helsinki in its sixth revision as well as the “International Council for Harmonization of Technical Requirements for Pharmaceuticals for Human Use–Good Clinical Practice (ICH-GCP)” guidelines. Safety of participants was supervised by an independent safety review committee. The study is registered with ClinicalTrials.gov, number NCT02237586.

### Data and statistics.

Data were captured on paper case report forms and entered into a database (OpenClinica, Waltham, MA; version 3.3) by independent double data entry. Data validation and statistical analysis were performed using R, version 3.3. Investigators, microscopists, and volunteers were blinded on hemoglobin status until Day 28. Blinding on malaria-exposure status was not practical because volunteers of the NI group were of European descent, and all others were Africans. Statistical analysis was performed using the log-rank test for time-to-event analysis and the Fisher’s exact test (2-tailed) for proportions, unless indicated otherwise.

## RESULTS

### Study population.

Twenty-five healthy volunteers, 12 women and 13 men between 18 and 28 years of age, were recruited of a group of 61 volunteers who were assessed for eligibility (Table 1). The five malaria-naive volunteers had resided in the study area for an average of 5 months (range: 1 week to 10 months). Genetic retyping of the 10 volunteers recruited into groups IA and IS revealed that one volunteer was mistyped by electrophoresis on screening as HbAS instead of HbAA. Hence, group IA consisted of 11 and group IS of nine volunteers (Supplemental Figure 1).

**Table 1:** Demographic characteristics of the volunteers

	Group NI	Group IA	Group IS
*N*	5	11	9
Males; females	1; 4	6; 5	6; 3
Caucasians; Africans	5; 0	0; 11	0; 9
Age in years (median, range)	28.0 (24.3–28.7)	22.1 (19.1–26.6)	22.5 (18.6–25.9)
Age in years (mean, SD)	26.8 (2.0)	21.9 (2.2)	22.2 (2.8)
Height in cm	173 (160–180)	165 (152–185)	167 (151–183)
Weight in kg	68 (61–70)	59 (46–75)	63 (40–75)
Body mass index in kg/m^2^	23.3 (18.9–26.6)	20.4 (18.8–27.0)	22.4 (16.2–23.6)

Median (range) for all continuous values, except where noted.

Four volunteers had asymptomatic parasitemia before clindamycin treatment. Three had gametocytemia, two by TBS and PCR, and another one by PCR only. After treatment with clindamycin, all volunteers were free of asexual parasites 1 day before CHMI (Day C-1).

### TBS positivity.

All (5/5) volunteers in group NI, 7/11 (64%) in group IA, and 5/9 (56%) in group IS were positive by TBS (Table 2). The difference in incidence of parasitemia between the NI group and IA and IS groups combined did not reach the level of statistical significance (*P* = 0.14).

**Table 2:** Parasitological results

	Group IA	Group IS	Group NI
Total number of subjects	11	9	5
Number of positive subjects by TBS	7 (64%)	5 (56%)	5 (100%)
Number of positive subjects by qPCR	9 (82%)	7 (78%)	5 (100%)
GM days to parasitemia by TBS, (range)	16.9 (13–24)	19.1 (15–25)	12.6 (12–14)
GM days to parasitemia by PCR (range)	7.9 (6–11)	10.6 (7–25)	7.9 (7–9)
GM days from PCR positivity to TBS positivity (range)	8.2 (5–16)	10.9 (7–17)	4.5 (3–5)
Number of positive subjects with symptoms through Day 28 (percentage among positives by TBS and among all)	6 (86%, 55%)	1 (20%, 11%)	5 (100%)
Number of positive subjects with symptoms or parasitemia > 1,000/μL through Day 28 (% among positives by TBS and among all positives)	6 (86%, 55%)	2 (40%, 22%)	5 (100%)
GM days to symptoms in those developing symptoms (range)	18.8 (16–25)	18.5 (18–19)	12.6 (12–14)
GM days from TBS positivity to symptoms in those developing symptoms (range)	1.8 (0–4)	1.3 (0–4)	0
GM days from PCR positivity to symptoms in those developing symptoms (range)	9.7 (5–17)	10.0 (9–11)	4.5 (3–5)
GM parasite density (/μL) at first parasitemia (range)	14 (7–67)	7 (3–14)	5 (2–9)

GM = geometric mean; PCR = polymerase chain reaction; qPCR = quantitative polymerase chain reaction; TBS = thick blood smear.

### qPCR positivity.

Two of the four TBS-negative volunteers in group IA and two of the four in the group IS were positive by qPCR. Thus, 9/11 volunteers in group IA and 7/9 volunteers in group IS were positive by qPCR (Table 2) during the 28-day follow-up. The difference in incidence of parasitemia between the NI group and IA and IS groups combined again did not reach the level of statistical significance (*P* = 0.55). Of note, all four volunteers who did not develop parasitemia were female (*P* = 0.01).

### Time to parasitemia by TBS and qPCR.

**Figure 1.**
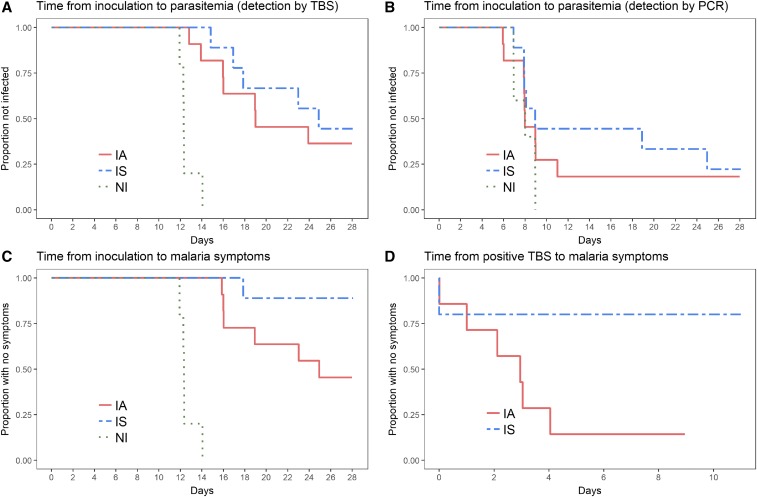
(**A**) Kaplan-Meier curve for time from inoculation to parasitemia detected by thick blood smear. (**B**) Kaplan-Meier curve for time from inoculation to parasitemia detected by PCR. (**C**) Kaplan-Meier curve for time from inoculation to malaria symptoms. (**D**) Kaplan-Meier curve for time from positive thick blood smear to malaria symptoms. This figure appears in color at www.ajtmh.org.

The geometric mean time from injection of PfSPZ Challenge until the first positive TBS was 12.6, 16.9, and 19.1 days for the NI, IA, and IS groups, respectively (ranges: 12 to 14, 13 to 24, and 15 to 25, respectively). The time to parasitemia by TBS for the NI group was significantly shorter than that for the IA (*P* = 0.016) and IS (*P* = 0.021) groups (Figure 1A). Times to parasitemia by qPCR were 7.9, 7.9, and 10.6 days for the NI, IA, and IS groups, respectively. Time to parasitemia detected by qPCR was similar between the three groups (log-rank test: *P* = 0.43) and between IA and IS groups (*P* = 0.47) (Figure 1B). There was no significant effect of sexual (*P* = 0.64) or asexual parasite (*P* = 0.46) carriage at screening on time to parasitemia.

### Parasite replication rates.

Parasite growth measured by qPCR in the NI group was consistent with previous trials in nonimmunes,^6,7^ which typically have shown an approximate 10-fold exponential multiplication every 48 hours from Day 7 on. By contrast, all lifelong malaria–exposed adults controlled, at least partially, parasite multiplication, although an actual rate could not be calculated because of the irregular patterns (Supplemental Figures 2 and 3). The pattern of parasitemia over time and the area under the curve were similar between groups IA and IS. There was a considerable variation that can be explained in part by technical problems during storage and extraction of nucleic acids. As a consequence, no parasites were detected in some samples that in reality had low parasitemia. Parasitemia associated with a spike appearing late in the follow-up was genotyped. All parasites were found to be identical to the CHMI strain.

### Clinical malaria or parasitemia > 1,000 parasites/μL blood by TBS.

The study protocol called for treatment of all volunteers in the NI group at the first detection of parasitemia, but treatment of IA and IS volunteers was given only if they had clinical malaria or asymptomatic parasitemia ≥ 1,000 parasites/μL by TBS. One volunteer in the IS group presented with parasitemia ≥ 1,000 parasites/μL (3,910/μL) without symptoms and was treated as per the protocol. All volunteers in the NI group, but only 6/11 volunteers in the IA group and 1/9 volunteers in the IS group, developed parasitemia and symptoms. The difference between NI and the IA and IS groups combined was significant (*P* = 0.003), but there was no difference between NI and IA groups (*P* = 0.12) (Figure 1C). The difference between IA and IS was significant (chi-squared test, *P* = 0.04).

In volunteers with clinical malaria, time between positive TBS and first malaria-attributed symptoms was different between malaria-naive (0 hours) and lifelong–malaria exposed adults, but it did not reach the level of statistical significance (Figure 1D; IA: 1.8, IS: 1.2 days; log-rank test: *P* = 0.09).

There was no significant effect of sexual (*P* = 0.49) or asexual parasite (*P* = 0.23) carriage at screening on time to clinical malaria.

### Antibodies response.

There was no association between antibody levels to PfCSP, PfEXP1, or PfMSP1 before CHMI and whether IA and IS subjects developed parasitemia by TBS. A total of 3 (15%), 9 (45%), and 9 (45%) of the 20 semi-immunes and 0, 1 (20%), and 2 (40%) of the five nonimmunes developed antibodies to PfCSP, PfEXP1, and PfMSP1, respectively (Table 3). Not surprisingly, the rate of development of antibodies to the blood-stage antigens (PfEXP1 and PfMSP1) was higher in semi-immunes who did as compared to those who did not develop parasitemia detected by TBS. For example, 9/12 (75%) and 0/8 (*P* = 0.0014) of volunteers who did and did not become parasitemic by TBS developed antibodies to PfEXP1, and 9/12 and 1/8 (*P* = 0.02) developed antibodies to PfMSP1 (Table 3).

**Table 3:** Number of individuals per group who developed antibodies to PfCSP, PfEXP1, and PfMSP1 between the day of CHMI and 28 days after CHMI

Group	TBS result	PfCSP	*P*	PfEXP1	*P*	PfMSP1	*P*
NI	Positive	0/5 (0%)	N/A	1/5 (20%)	N/A	2/5 (40%)	N/A
IA	Negative	0/4 (0%)	–	0/4 (0%)	–	0/4 (0%)	–
	Positive	3/7 (43%)	0.24	5/7 (71%)	0.06	5/7 (71%)	0.06
IS	Negative	0/4 (0%)	–	0/4 (0%)	–	0/4 (0%)	–
	Positive	1/5 (20%)	1.00	4/5 (80%)	0.048	4/5 (80%)	0.048
IA plus IS	Negative	0/8 (0%)	–	0/8 (0%)	–	0/8 (0%)	–
	Positive	4/12 (33%)	0.12	9/12 (75%)	0.001	9/12 (75%)	0.001

CHMI = controlled human malaria infection; PfCSP = *Plasmodium falciparum* (Pf) circumsporozoite protein; PfEXP1 = Pf exported protein-1; PfMSP1 = Pf merozoite surface protein-1; TBS = thick blood smear. Those who developed parasitemia had significantly greater rates of seroconversion to PfEXP1 and PfMSP1 than those who did not.

Semi-immune subjects who had parasitemia with symptoms had a more pronounced increase in antibodies against all three antigens from baseline to Day 28 compared with those with asymptomatic parasitemia (Wilcoxon test comparing the ratio OD 1.0 on Day 28 to before CHMI (Day 0) for symptomatic versus asymptomatic individuals: PfCSP: 1.0 versus 1.7, *P* = 0.046; PfEXP1: 0.96 versus 41, *P* = 0.003; PfMSP1: 1.3 versus 52, *P* = 0.04) (Supplemental Figure 4).

### AEs.

Injection of PfSPZ Challenge was well tolerated, and no serious AE occurred during the trial. Every volunteer had at least one AE, and a total of 470 AEs were recorded over 208 days of observation.

**Figure 2.**
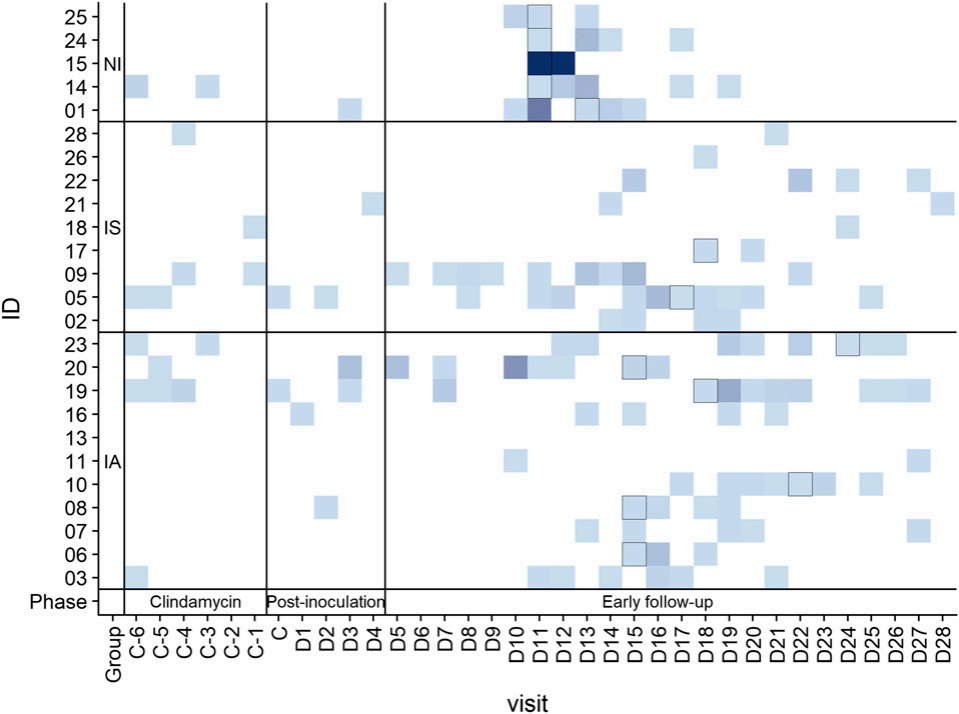
Heatmap showing intensity of adverse events over time. The color gradient shows number of events with deeper blue signifying more events than light blue. The days of first appearance of symptoms are indicated with a black frame. This figure appears in color at www.ajtmh.org.

Pretreatment with clindamycin was well tolerated with 21 mild events in 10 volunteers. During the 5 days after injection of PfSPZ Challenge, there was one related local reaction (injection site pruritus) and four systemic events (two nausea, one diarrhea, one fatigue). Between 6 and 28 days after injection of PfSPZ Challenge, there were 314 AEs recorded in this period, of which 224 (71%) were related to malaria. Malaria-related symptoms were more severe in nonimmunes (Figure 2), but no other differences between study groups were found (Table 4).

**Table 4:** Number of volunteers having trial-related events over the whole trial, segregated by severity

	Group IA* (*N* = 11)		Group IS* (*N* = 9)		Group NI (*N* = 5)		
	Grade 1	Grade 2	Grade 1	Grade 2	Grade 1	Grade 2	Grade 3
Headache	9 (82%)	3 (27%)	6 (67%)	4 (44%)	3 (60%)	3 (60%)	0
Fatigue	9 (82%)	1 (9%)	4 (44%)	0	4 (80%)	4 (80%)	2 (40%)
Myalgia	6 (55%)	3 (27%)	3 (33%)	1 (11%)	2 (40%)	2 (40%)	0
Nausea	6 (55%)	1 (9%)	5 (56%)	0	3 (60%)	1 (20%)	0
Arthralgia	5 (45%)	2 (18%)	2 (22%)	1 (11%)	1 (20%)	0	2 (40%)
Diarrhea	6 (55%)	0	5 (56%)	0	2 (40%)	0	0
Pyrexia	2 (18%)	2 (18%)	2 (22%)	1 (11%)	2 (40%)	2 (40%)	2 (40%)
Chills	3 (27%)	0	2 (22%)	0	2 (40%)	0	3 (60%)
Vomiting	3 (27%)	1 (9%)	3 (33%)	0	1 (20%)	1 (20%)	0
Dizziness	4 (36%)	0	3 (33%)	0	1 (20%)	0	0
Abdominal pain	3 (27%)	0	2 (22%)	0	1 (20%)	0	0
Platelet count abnormal	3 (27%)	0	2 (22%)	0	0	0	0
Palpitations	2 (18%)	0	0	0	2 (40%)	0	0
Hemoglobin abnormal	1 (9%)	0	2 (22%)	0	0	0	0
Leukocyte count abnormal	2 (18%)	0	1 (11%)	0	0	0	0
AST abnormal	2 (18%)	0	0	0	0	0	0
Chest pain	1 (9%)	0	1 (11%)	0	0	0	0
Dysgeusia	0	0	2 (22%)	0	0	0	0
Dyspnea	1 (9%)	0	0	0	0	0	1 (20%)
ALT abnormal	1 (9%)	0	0	0	0	0	0
Asthenia	1 (9%)	0	0	0	0	0	0
Blood creatinine abnormal	0	0	0	0	1 (20%)	0	0
Decreased appetite	0	0	1 (11%)	0	0	0	0
Injection site pruritus	0	0	1 (11%)	0	0	0	0
Lymphocyte count abnormal	0	0	1 (11%)	0	0	0	0
Neutrophil count abnormal	1 (9%)	0	0	0	0	0	0
Rash	0	0	0	0	1 (20%)	0	0
Skin irritation	0	0	0	0	1 (20%)	0	0
Tachycardia	0	0	1 (11%)	0	0	0	0
Tachypnea	1 (9%)	0	0	0	0	0	0

* No grade 3 trial related adverse events were recorded for groups IA and IS.

Of 1,663 laboratory measurements, 130 (8%) were out of range, but none were considered as clinically significant. Seven of these were grade 3 on the toxicity scale (four cases of low thromboyctes and one case of low leukocytes, lymphocytes, or neutrophils each, see Supplement Table 1). Of these, five occurred during a malaria episode, and all normalized in the course of follow-up.

## DISCUSSION

In this study, we observed that all malaria-experienced volunteers controlled parasitemia; some manifested sterile protection, others exponential growth but with lower parasite multiplication rates compared with malaria-naive volunteers. The ability to control parasitemia was shown most strikingly by the differences between the time to parasitemia of the five nonimmune volunteers and that of the volunteers with lifelong exposure. Furthermore, all five nonimmunes developed clinical malaria, whereas only seven of 20 malaria-exposed volunteers did (*P* = 0.015).

Two studies using PfSPZ Challenge have been performed in African adults and have been published.^21,22^ In these studies, volunteers were recruited in a low-endemicity area and were inoculated using intradermal or intramuscular injection. Intradermal inoculation did not lead to parasitemia in 100% of recipients even in malaria-naive adults,^23,24^ whereas intramuscular injection of at least 75,000 PfSPZ showed similar infection rates and parasite kinetics as DVI of 3,200 PfSPZ.^6,7^ The latter regimen has become the gold standard for CHMI studies with PfSPZ Challenge, and to date 63 of 63 (100%) volunteers with no or minimal malaria exposure have become parasitemic.^6,7^ Before that, CHMI studies in lifelong malaria–exposed subjects were conducted using locally infected mosquitoes, preparations thereof (e.g., fresh salivary gland preparations for parenteral inoculation) and blood from donors with high asexual parasitemia.^3,5,9^ In some cases, these methods were combined. By contrast, PfSPZ Challenge, similar to Sanaria PfSPZ Vaccine, which is the same product but radiation attenuated,^15^ is derived from a master cell bank and produced according to good manufacturing practice guidelines. Therefore, volunteers are infected with exactly the same material and, when given by DVI, using a tightly controlled dose, an approach that cannot be reproduced using mosquitoes. Consequently, expression patterns of immunologically variant antigens, i.e., var genes in malaria-naive volunteers, are conserved,^25,26^ a prerequisite to understand naturally acquired immunity.

Resistance to severe and complicated malaria in lifelong-exposed adults has been investigated for more than 100 years^8^ and serves as a main argument for developing asexual blood stage vaccines. The strongest evidence for naturally acquired immunity (NAI) after lifelong exposure comes from passive transfer experiments using antibody preparations from lifelong malaria–exposed individuals to treat malaria patients with no or low-level immunity,^27–29^ but there are recently minimal data on how NAI influences infection kinetics using modern clinical trial and diagnostic methodology.

To our knowledge, this is the first time that naturally acquired sterile protection has been confirmed using highly sensitive diagnostic methods, and that patterns of parasite control have been characterized in semi-immune subjects. One can only speculate how the four volunteers achieved sterile protection; potential mechanisms include 1) prevention of infection of hepatocytes, 2) elimination of infected liver cells by direct T cell–mediated cytotoxicity or by immune mediators (e.g., interferon-γ and nitric oxide), 3) highly effective clearance of the first generation of merozoites leaving the infected hepatocyte, or a combination of these factors. In this study, antibodies to PfCSP, PfEXP1, or PfMSP1 before CHMI were not associated with development or lack of development of parasitemia. Therefore, we assume that a combination of the above-mentioned adaptive immune mechanisms is the most likely reason for the differences in time to parasitemia between nonimmunes and semi-immunes. Because no method to measure liver load in humans is available, it will be challenging to completely elucidate the mechanism of naturally acquired sterile immunity. Other insights may come from in-depth immunology analyses currently being performed with samples from this study. These will be published separately.

More than 60 years ago, CHMI was used to study the effect of NAI and sickle cell trait on malaria. One study showed a high rate of protection in sickle cell trait carriers from Uganda compared with subjects with normal hemoglobin,^5^ but all subsequent trials with malaria-naive^4^ and lifelong-exposed subjects^3^ showed no significant effect of sickle cell trait on infection rate and early parasite kinetics. Our data are in agreement with the latter studies showing no large effect of sickle cell trait on probability of infection; however, a significant difference was observed in occurrence of disease. Only one of the nine volunteers with sickle cell trait developed clinical malaria. This is corroborated by our recent data from a vaccine trial in 34 semi-immune subjects with subsequent CHMI (B. Lell et al., unpublished data). Aggregating both studies showed a significant effect of HbAS in preventing symptoms with 20/40 volunteers in the IA groups combined developing parasitemia with symptoms compared with 2/14 in the IS groups (*P* = 0.027).

Apart from the well-described effect of sickle cell trait on morbidity and mortality, epidemiological data on time-to-reinfection as a measure of infectivity under natural exposure in different transmission settings are also inconsistent, ranging from no effect of hemoglobin genotype on time to reinfection^12^ to a nonsignificant trend of longer reinfection times in HbAS individuals^30^ to a strong effect of increased time to reinfection.^31,32^ Some of this discrepancy may be explained by characteristics of the sample and end points. Although CHMI with PfSPZ is the most stringent approach to address this issue, the inoculum dose may have an important effect on infection rate and is likely to be lower under natural exposure compared with CHMI. Our study showed somewhat longer times to develop parasitemia in those with sickle cell trait, suggesting that a repeat study with a larger sample size might be instructive.

In summary, our data show that even with small sample sizes, CHMI with PfSPZ Challenge can be used to gain insight into the impact of NAI and innate factors (e.g., sickle cell trait) on incidence of infection with Pf and symptoms associated with infection. NAI prolonged the time to parasitemia, and sickle cell trait may have prolonged it further. NAI alone did not have a significant impact on the incidence of symptoms associated with parasitemia, but the combination of NAI and sickle cell trait significantly reduced the clinical manifestations of Pf malaria.

## Supplementary Material

Supplemental Figure and Table.
