# Supplementary material for: Impact of Sickle Cell Trait and Naturally Acquired Immunity on Uncomplicated Malaria after Controlled Human Malaria Infection in Adults in Gabon
Source: Am J Trop Med Hyg. 2017 Dec 18;98(2):508–15. doi: 10.4269/ajtmh.17-0343 (PMC5929186; doi:10.4269/ajtmh.17-0343)
Supplement: Supplementary file 1 [file tpmd170343.SD1.pdf]

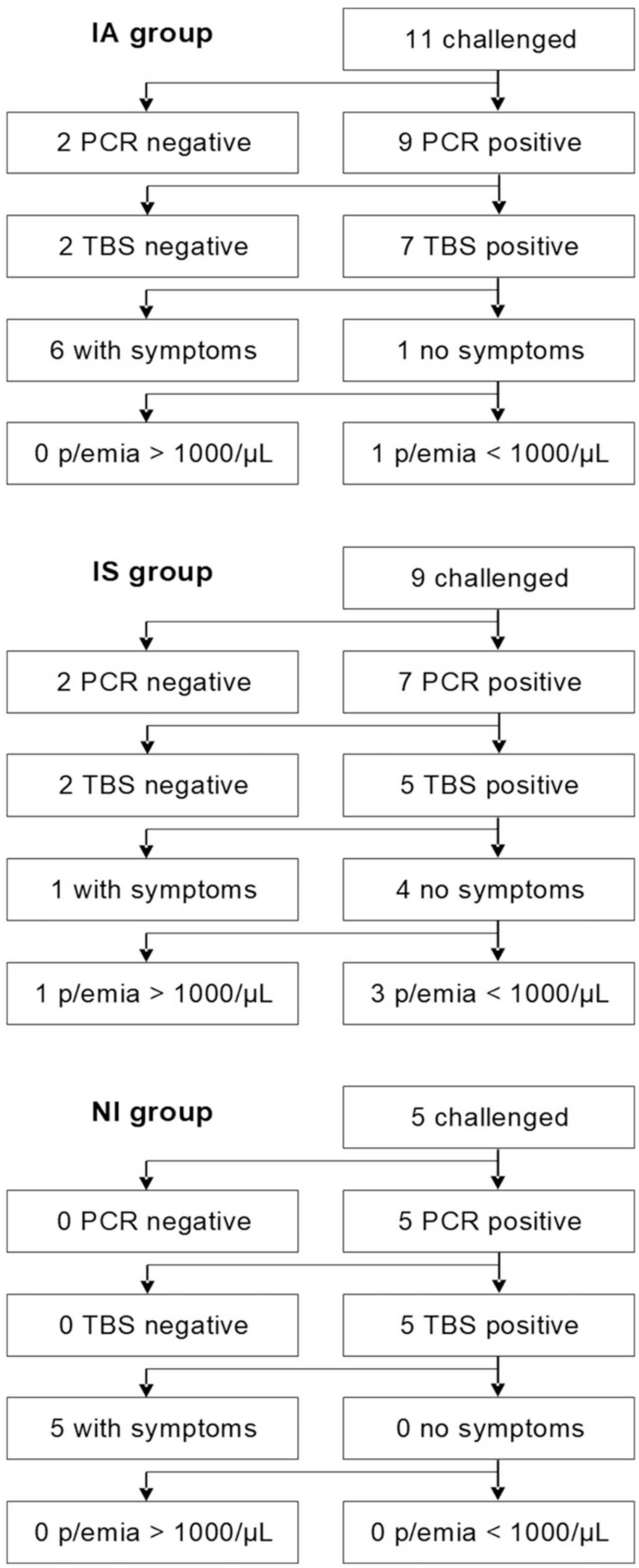

SUPPLEMENTAL FIGURE 1. Subject flow diagram.

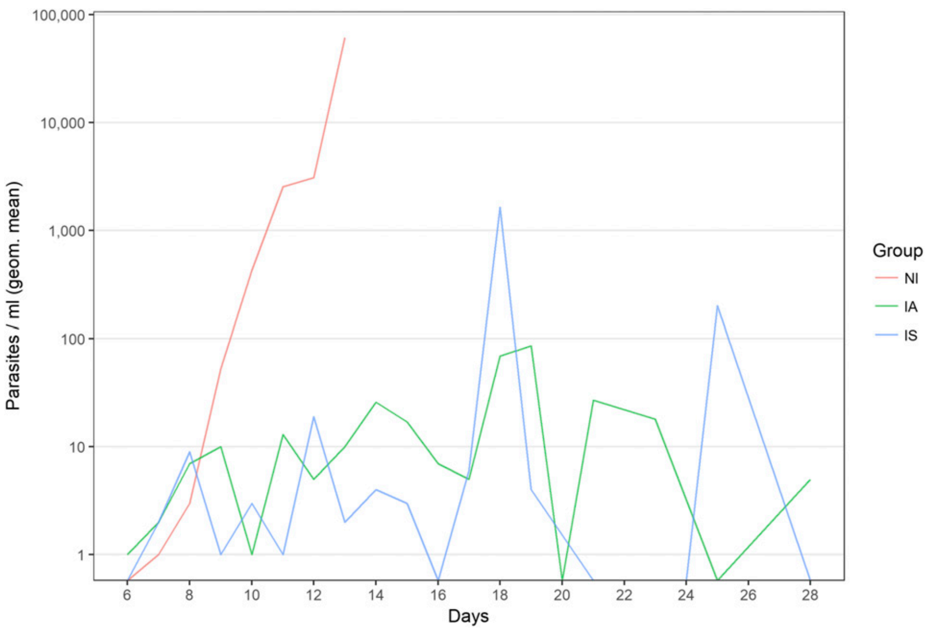

Supplemental Figure 2. Geometric mean parasitemia per milliliter on a logarithmic scale over the study, segregated by study group. Because of technical problems during storage and extraction of nucleic acids, the detection limit was raised for some samples. Samples with low parasitemia might therefore have incorrectly been recorded as negative.

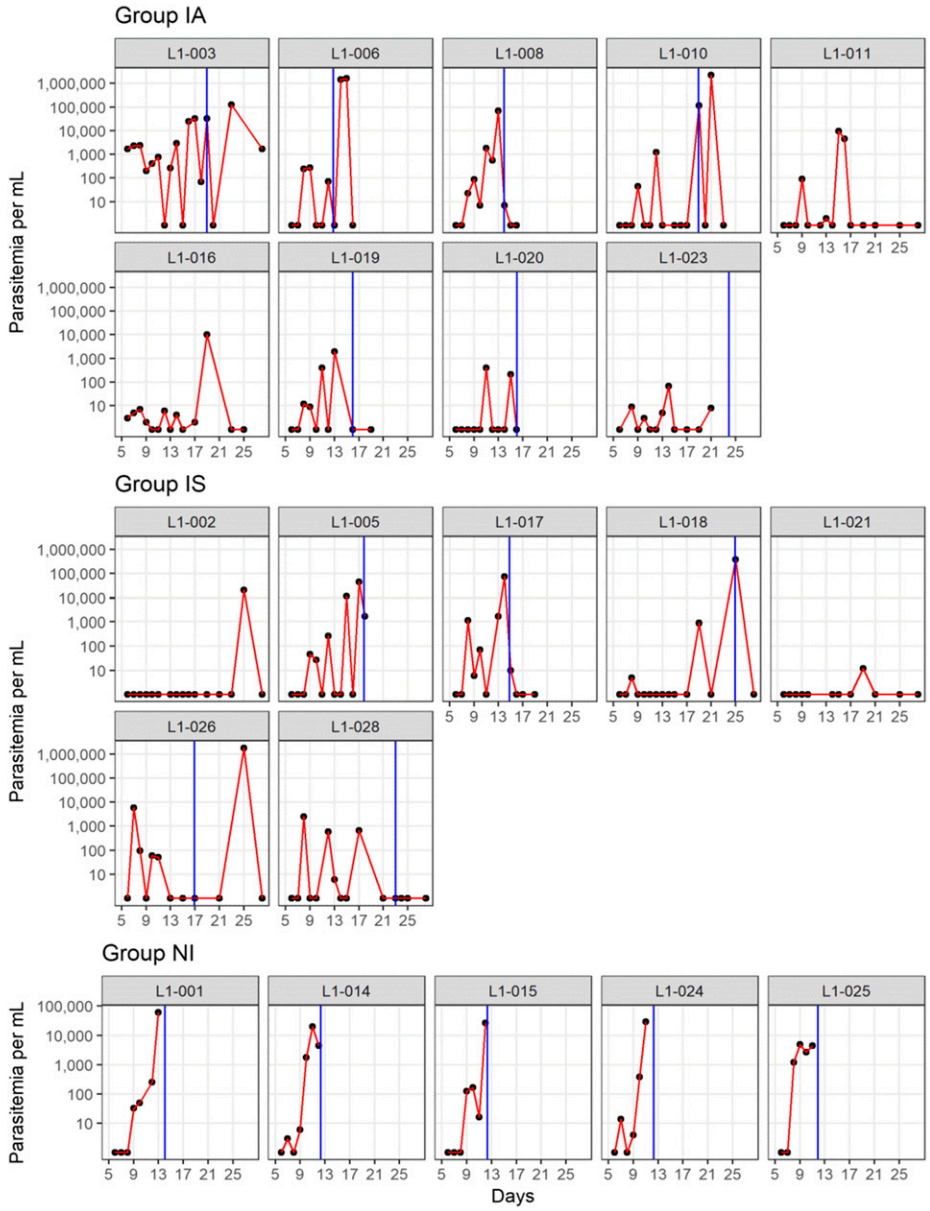

Supplemental Figure 3. Course of parasitemia over the study for each individual, grouped by study group. Vertical blue lines indicate time of thick blood smear positivity. Because of technical problems during storage and extraction of nucleic acids, the detection limit was raised for some samples. Samples with low parasitemia might therefore have incorrectly been recorded as negative.

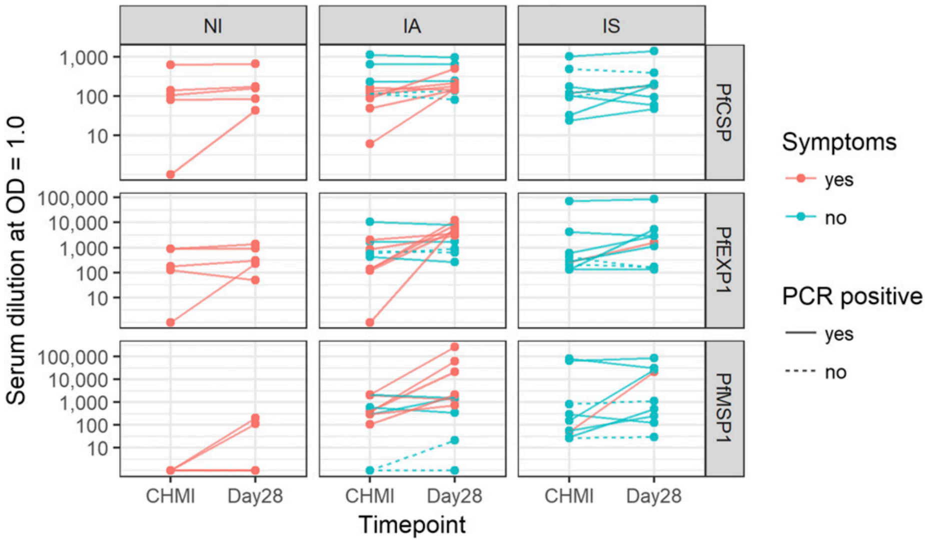

Supplemental Figure 4. Antibodies (serum dilution at which the optical density [OD] was 1.0) against *Plasmodium falciparum* circumsporozoite protein (PfCSP), exported protein-1 (PfEXP1) and merozoite surface protein-1 (PfMSP1) at the time of controlled human malaria infection (CHMI) and 28 days later.

Supplement Table 1

Grade 3 laboratory adverse events (AEs)

Subject ID	Group	Visit*	Sex	AE	Value (/nL)	Grade 3 limit (/nL)
L1-005	IS	Malaria	F	Leukocytes decreased	1.2	< 1.5
L1-005	IS	Malaria	F	Lymphocytes decreased	0.33	< 0.5
L1-005	IS	Malaria	F	Neutrophils decreased	0.84	< 1.0
L1-005	IS	Malaria	F	Thrombocytes decreased	91	< 100
L1-016	IA	Day 84	M	Thrombocytes decreased	91	< 100
L1-017	IS	Malaria	M	Thrombocytes decreased	96	< 100
L1-023	IA	C-1	F	Thrombocytes decreased	97	< 100

* "Malaria" is during an episode of malaria; "C-1" is the day before challenge.
